# Pediatric in-hospital emergencies: real life experiences, previous training and the need for training among physicians and nurses

**DOI:** 10.1186/s13104-019-4051-4

**Published:** 2019-01-14

**Authors:** Ronny Lehmann, Anke Seitz, Jochen Meyburg, Bettina Hoppe, Georg Friedrich Hoffmann, Burkhard Tönshoff, Sören Huwendiek

**Affiliations:** 1 Department of Pediatrics I, Center for Pediatrics and Adolescent Medicine, Im Neuenheimer Feld 430, 69120 Heidelberg, Germany; 2 Department for Assessment and Evaluation, Institute for Medical Education, Mittelstrasse 43, 3012 Bern, Switzerland

**Keywords:** Pediatrics, Emergency medicine, Interprofessional education, Needs assessment, Continuing medical education

## Abstract

**Objective:**

Pediatric emergencies challenge professional teams by demanding substantial cognitive effort, skills and effective teamwork. Educational designs for team trainings must be aligned to the needs of participants in order to increase effectiveness. To assess these needs, a survey among physicians and nurses of a tertiary pediatric center in Germany was conducted, focusing on previous experience, previous training in emergency care, and individual training needs.

**Results:**

Fifty-three physicians and 75 nurses participated. Most frequently experienced emergencies were respiratory failure, resuscitation, seizure, shock/sepsis and arrhythmia. Resuscitations were perceived as being particularly precarious. Team collaboration and communication were major issues arising from previous emergency situations, but perceptions differed between physicians and nurses. Regarding previous training, physicians were accustomed to self-directed learning, whereas nurses usually attended practical courses. Both physicians and nurses rated themselves as having moderate levels of knowledge and skills for pediatric emergencies, though residents reported the significantly lowest preparedness. Both professions reported a high need for training of basic procedures and emergency algorithms, physicians even more than nurses.

## Introduction

Several studies have revealed major deficits in emergency preparedness among professional staff [1–6]. Most serious errors in critical care occur because of poor communication or collaboration, rather than individual mistakes [7, 8]. For example, one prospective observational study by Hunt et al. conducted pediatric emergency simulations with mock codes. Of the teams involved, 75% deviated from current resuscitation guidelines, and all demonstrated communication errors [9].

The importance of team training to improve team behavior is more and more highlighted [7] using simulation-based training as standard educational approach [10–12]. Team training allows shared expertise and perspectives based on group knowledge and skills [13]. On-site simulation training can massively increase opportunities for learning for local staff when embedded in the hospital routine [14]. Educational strategies need to be optimized in such interprofessional training programs to improve patient outcomes [1, 7].

Little is known about whether previous experiences with emergencies, previous training, and individual needs for pediatric emergency training differ between physicians and nurses or how these should be taken into account when developing interprofessional team training. Therefore, we surveyed the physicians and nurses of a tertiary pediatric center in Germany on these issues as the first step in developing an in-house team training program. For this needs assessment, our goal was to identify frequent and/or critical in-house emergency situations, and aspects which support or impede them. Further we wanted to know how educational strategies differed between physicians and nurses, in order to appraise acceptance for different teaching methods that could be used. Also, concrete training objectives (procedures, algorithms) needed to be determined by assessing individual demands.

We hypothesized similar experiences with emergencies by both, physicians and nurses, but with differing perspectives depending on professional roles. We expected acceptance for self-directed learning as limited especially for a non-academic profession like nursing, and the available time for additional hands-on courses as limited in particular among physicians. Furthermore, we expected comparable high needs for training of basic as well as advanced procedures among medical and nursing staff.

## Main text

### Methods

#### Participants

We invited all the physicians and nurses of the tertiary Center for Pediatrics and Adolescent Medicine, Heidelberg, Germany, to participate in this survey. At the time of the survey which was conducted in autumn 2010, 127 physicians and 410 nurses were employed. Participation was voluntary and anonymous.

#### Evaluation instruments

The survey was structured into four sections: basic data of the participants, previous experience of in-house emergencies, previous training on emergencies, and individual needs for such training. For details of used items see Table 1. Questions were either open, allowing multiple answers, or requested agreements on a 5-point Likert scale from 1 (totally disagree) to 5 (totally agree). Selections of training needs were taken from current guidelines [15], published questionnaires on pediatric emergency preparedness [16–20], and interviews with in-house experts. The survey forms were pilot-tested with physicians and nurses in think-aloud studies before implementation, and revisions were made to ensure clarity and response process validity [21]. We used a web-based survey platform, as well as paper-based survey forms distributed to all possible participants. Where appropriate, the nurse-directed survey form asked for assistance in the procedures as some procedures are only performed by physicians (especially venipuncture is a physician activity at the study site). Trauma management was not addressed, as this is provided by the local pediatric surgery department.

**Table 1:** Survey form and numerical results

	Item	Medical staff	Nursing staff	
		N = 53 (41.7% of medical house staff)	N = 75 (18.3% of nursing house staff)	
Basic data	Age (years)	32.7 ± 4.8	36.2 ± 11.6	
	Gender	65.8% female 34.2% male	96.4% female 3.6% male	
	Clinical practice (years)	8.2 ± 7.0	15.3 ± 10.7	
	Level of qualification	(Resident, specialist or consultant)	(Nurse or head nurse)	
Previous experience	Which critical or emergency situations did you experience so far in the hospital?	(Free text)	(Free text)	
	In which of them did you feel the most insecure?	(Free text)	(Free text)	
	What went good in these situations?	(Free text)	(Free text)	
	What could have been better in these situations?	(Free text)	(Free text)	
Previous training	How did you acquire knowledge about managing emergencies so far?	(Free text, Fig. 1)	(Free text, Fig. 1)	
	How did you acquire practical skills (like bag-valve-mask ventilation, intubation etc.) so far?	(Free text, Fig. 1)	(Free text, Fig. 1)	
Individual needs assessment	I have adequate knowledge to handle pediatric emergencies	(Figure 2)	(Figure 2)	
	I have adequate skills to handle pediatric emergencies	(Figure 2)	(Figure 2)	
	I need to have more training on…			*p*-*value*
	Pediatric basic life support	4.8 ± 0.5	4.5 ± 0.8	0.011
	Pediatric advanced life support	4.5 ± 0.7	4.2 ± 0.9	0.119
	Bag-valve-mask ventilation	4.8 ± 0.5	4.4 ± 0.9	0.002
	Endotracheal intubation	4.3 ± 0.8	3.7 ± 1.2	0.005
	Intravenous access	4.1 ± 1.3	3.2 ± 1.4	< 0.001
	Central venous access	3.8 ± 1.2	3.4 ± 1.2	0.065
	Intraosseous access	3.9 ± 1.2	3.3 ± 1.3	0.005
	Newborn life support	4.6 ± 0.8	3.4 ± 1.3	< 0.001
	Management of foreign body aspiration	4.7 ± 0.6	4.1 ± 1.0	< 0.001
	Management of unconsciousness	4.7 ± 0.6	4.2 ± 0.9	< 0.001
	Management of respiratory distress	4.7 ± 0.7	4.0 ± 1.0	< 0.001
	Management of anaphylactic reactions	4.6 ± 0.8	4.2 ± 0.9	0.004
	Management of seizures	4.7 ± 0.7	4.0 ± 1.0	< 0.001
	Management of arrhythmia	4.5 ± 0.7	3.8 ± 1.2	0.001
	Management of shock	4.6 ± 0.7	4.2 ± 0.9	0.008
	Management of metabolic emergencies	4.4 ± 0.8	3.2 ± 1.3	< 0.001
	Team communication	4.3 ± 0.9	4.0 ± 1.1	0.167
	Which objectives for training are missing?	(free text)	(free text)	
	I would like to participate in an interprofessional course (physicians and nurses) on pediatric emergencies.	4.7 ± 0.6	4.6 ± 0.8	0.443

Mean ± standard deviation or percentages. Agreements in the individual needs assessment on Likert scale from 1 (totally disagree) to 5 (totally agree)

#### Data analysis

Free-response answers in the sections on previous emergency experiences and previous training were analyzed using content analysis; keywords were determined in order to assess frequencies of answer categories [22]. Previous training was compared using Chi square tests for comparable answer categories. Self-assessed levels of adequate knowledge and skills on pediatric emergencies were compared using a one-factor analysis of variance (ANOVA) with the between-subject factor ‘profession and qualification level’ and the dependent variables ‘knowledge’ and ‘skills’. Least significant difference (LSD) post hoc tests were conducted where appropriate, including Bonferroni corrections for multiple comparisons. Statistical differences for individual training needs were calculated using t-tests for between-group comparisons. IBM SPSS Statistics Version 24 (IBM Corporation, Armonk, NY) was used for analysis, with an α level of 0.05.

Numerical results are given as mean ± standard deviation, or percentages. For open questions, the five most frequent responses are presented, followed by their percentage of all given answers in brackets.

### Results

#### Participation and basic data

Twenty-six residents, 12 specialists and 15 consultants participated in the survey (together 41.7% of the center’s medical staff), as well as 64 nurses and 11 head nurses (18.3% of the nursing staff), see Table 1. The mean ages of nurses and physicians were comparable (36.2 ± 11.6 vs. 32.7 ± 4.8 years); however, the mean duration of clinical practice of nurses was almost twice as long as that of physicians (15.3 ± 10.7 vs. 8.2 ± 7.0 years).

##### Experienced critical or emergency situations

Physicians, 92 responses:

1. Respiratory failure (21.7%);
2. Resuscitation (14.1%);
3. Seizure (14.1%);
4. Shock and sepsis (7.6%) and arrhythmia (7.6%).

Nurses, 117 responses:

1. Respiratory failure (31.6%);
2. Resuscitation (20.5%);
3. Seizure (12.8%);
4. Shock and sepsis (11.1%);
5. Arrhythmia (10.3%).

##### Situations feeling the most insecure

Physicians, 37 responses:

1. Resuscitation (32.4%);
2. Airway and respiration management (24.3%);
3. Management of arrhythmia (13.5%);
4. Management of circulation issues other than arrhythmia (10.8%);
5. Newborn care (5.4%) and situations with lack of support by experienced colleagues (5.4%).

Nurses, 37 responses:

1. Situations with lack of support by physicians (27.0%);
2. Resuscitation (24.3%);
3. Teamwork with inexperienced physicians (21.6%);
4. Respiratory failure (16.2%);
5. Arrhythmia (5.4%).

##### What went good during previous emergencies

Physicians, 35 responses:

1. Teamwork between medical and nursing staff (51.4%);
2. Teamwork with the intensive care unit (ICU) team (25.7%);
3. Support by consultants (17.1%);
4. Team communication (2.9%);
5. Sufficient in-house emergency structures (2.9%).

Nurses, 75 responses:

1. Teamwork between medical and nursing staff (60.0%);
2. Distinct delegation of tasks and/or roles during emergency situations (14.7%);
3. Teamwork with the ICU team (12.0%);
4. Sufficient coordination within emergency situations (9.3%);
5. Sufficient in-house emergency structures (2.7%).

##### What could have been better during previous emergencies

Physicians, 38 responses:

1. Own lack of preparedness (39.5%);
2. Deficient in-house emergency structures (28.9%);
3. Deficient team communication (18.4%);
4. Lack of experienced support (7.9%);
5. Deficient equipment (5.3%).

Nurses, 34 responses:

1. Deficient coordination within emergency situations (41.2%);
2. Own lack of preparedness (23.5%);
3. Deficient team communication (11.8%);
4. Lack of experienced support (11.8%);
5. Deficient equipment (11.8%).

#### Previous training

**Fig. 1:**
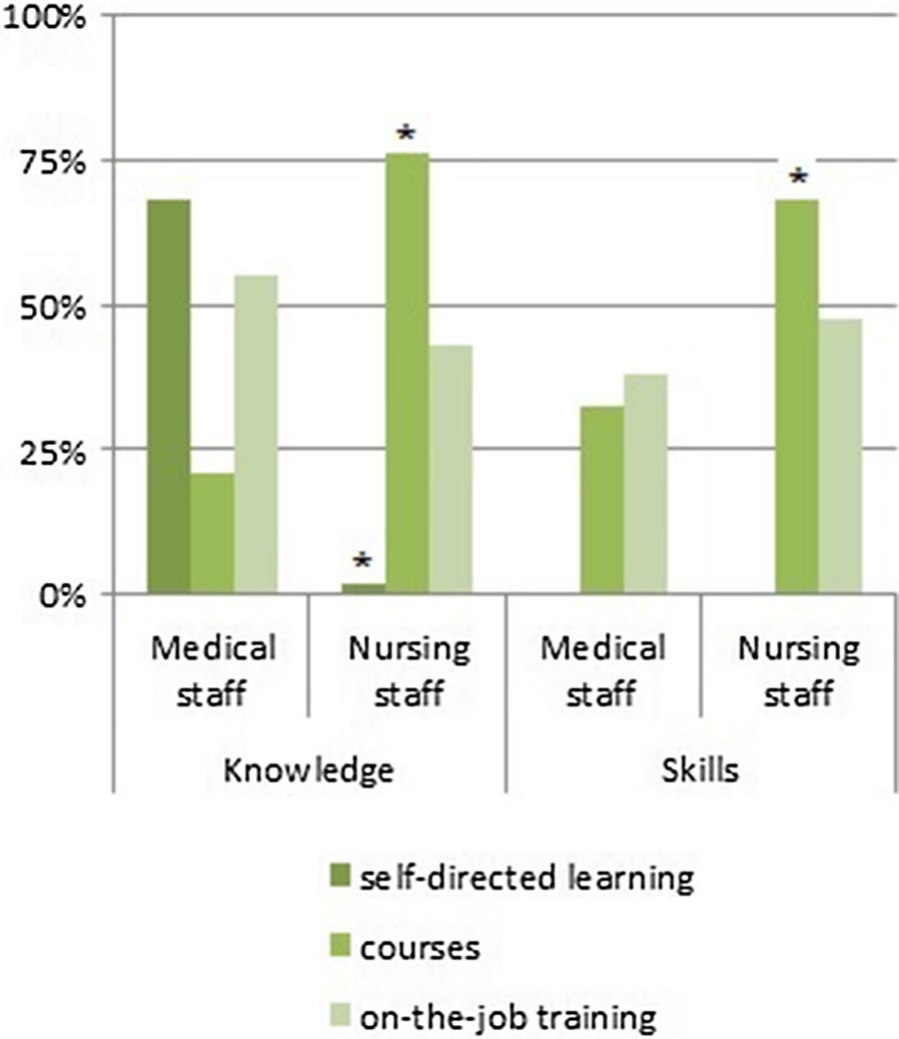
Educational behavior for acquiring knowledge and skills in pediatric emergencies (multiple answers possible). *p < 0.05 vs. medical staff

Self-directed learning played the major role in knowledge acquisition for physicians, whereas it was negligible for nurses, see Fig. 1. Attendance of practical courses for acquiring skills related to pediatric emergencies was low among physicians; only 32% had previously attended one or more courses. Course attendance was distinctly more common among nurses for acquiring both knowledge and skills (76% and 68%, respectively). On-the-job training for knowledge and skill acquisition was important for both medical and nursing staff.

#### Individual needs assessment

**Fig. 2:**
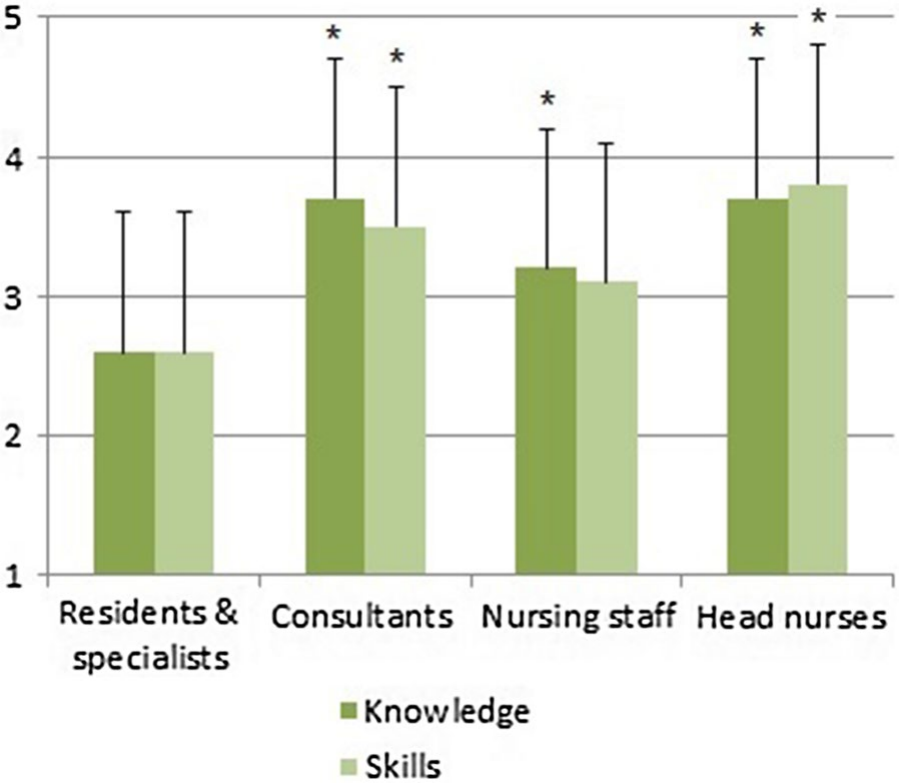
Agreements for having adequate knowledge and skills concerning pediatric emergencies. Agreements on Likert scale from 1 (totally disagree) to 5 (totally agree); *p < 0.05 vs. residents and specialists

Agreements to a certain level of knowledge and skills differed significantly concerning pediatric emergencies, see Fig. 2 (knowledge *F*(3,123) = 6.647, p < 0.001; skills *F*(3,123) = 5.832, p = 0.001). For individual training needs, see Table 1. Furthermore, physicians mentioned traumatology (two answers) as being an additional training need.

### Discussion

Physicians and nurses similarly ranked types of emergencies, and particularly communication and collaboration issues were perceived as either positive or problematic. Educational strategies differed distinctly between professions; nurses did often attend practical courses, whereas physicians tended to self-directed learning. Self-assessed knowledge and skills for pediatric emergencies were moderate for both, medical and nursing staff, but slightly higher among nurses. Self-identified individual training needs were high for both physicians and nurses, especially for basic procedures and frequently required emergency algorithms.

Both physicians and nurses reported the same types of emergencies as most frequently experienced. This is comparable to the pediatric emergency prevalence reported by others [23, 24]. Resuscitation situations were judged the most precarious for both professions. Remarkably, resuscitation happens rarely on peripheral wards outside of the ICU, but has obviously left strong impressions.

Physicians reported more frequently than nurses that their own emergency preparedness caused feelings of insecurity. In contrast, nurses mentioned aspects of teamwork as the most frequent cause of feeling insecure. These responses might be related to differences in clinical experience between medical and nursing staff; the nurses in this survey had significantly more clinical experience. Teamwork and communication are key factors in successfully managing emergency situations [7, 8], and this assertion was supported by our results; both physicians and nurses reported positive experiences regarding teamwork in previous emergency situations. Well-organized coordination and a clear distribution of roles in emergency situations appear to be important, especially for nurses [8].

The previous training of physicians and nurses differed considerably in our study. Physicians frequently used self-directed learning, which was unusual among nurses. Typically, physicians make active choices in how and what to learn from a diversity of offers, as a part of continuing medical education, rather than using standardized programs [25]. In contrast, nurses frequently attended practical courses. Wisniewski et al. found that face-to-face sessions and online web-courses were the preferred educational methods for nurses [19]—both guided forms of learning.

For self-reported preparedness, participants reported moderate levels of knowledge and skills for pediatric emergencies, with the lowest ratings by residents. This is comparable with other evaluation findings in residency programs [20, 26] and it is worth mentioning that residents tend to have lower self-confidence in their skills and knowledge than nurses [27]. However, high confidence does not necessarily lead to sufficient performance in real emergency encounters, as shown by Nadel et al. [3]. In our study, there were no significant differences in self-rated knowledge and skills within all professional groups.

When discussing their training needs in more detail, participants broadly agreed that training in procedures and algorithms is required; this is, again, similar to other reports [18, 28–30]. Both, physicians and nurses, reported training in basic procedures, such as pediatric basic life support, bag-valve-mask ventilation and most emergency algorithms as of the highest need. The greatest differences in training needs between physicians and nurses were for intravenous access, newborn life support and metabolic emergencies, which were all considered of high need by physicians but not nurses. As described above, venous access is a physician activity at the study site. In addition, most nurses do not work in specialized neonatal wards, and physicians usually have neonatal placements during their residencies. Metabolic emergencies are uncommon at most peripheral wards; they are more common at specialized wards and ICUs, and can be more often encountered by physicians during their residency placements. Both physicians and nurses identified team communication as core aspect to their emergency training.

To achieve pediatric emergency preparedness, many different approaches have been described, but often without evaluation of their short- and long-term outcomes [31]. Recent data highlight the value of simulation-based training for teams in acquiring technical and non-technical skills [12, 28, 32, 33]. Therefore small-group teaching is necessary and provides a controlled environment in which repetitive practice with close supervision is supported [34–36]. However, this type of training is expensive in terms of equipment and human resources [6]. To increase the effectiveness and efficiency of such training, a blended learning approach might be promising, with tailored preparation for each profession. There are positive reports on using such blended methods to teach clinical procedures to undergraduates [37]. Such an approach may specifically address the different learning behaviors and needs of nurse and physician groups, and subsequently enhance acceptance. It may also sensitize for inter-professional collaboration with somewhat differing learning objectives between professional groups.

### Conclusions

Differences of profession-specific roles and requirements, as well as in learning behaviors must be taken into account when developing interprofessional team trainings.

## Limitations

The data analysis is limited by the use and comparison of means of actually ordinal scaled results (Likert) and so only providing exploratory insights to existing differences between professions.

The given results reflect the subjective perceptions of the staff of one tertiary pediatric center in Germany; thus, they should be interpreted with caution and may not be fully applicable to other pediatric emergency care sites.

## Abbreviations

ANOVA: analysis of variance
ICU: intensive care unit
LSD: least significant difference
